# Crosstalk between Tumor and Stromal Cells in Pancreatic Ductal Adenocarcinoma

**DOI:** 10.3390/ijms21155486

**Published:** 2020-07-31

**Authors:** Nadine Sperb, Miltiadis Tsesmelis, Thomas Wirth

**Affiliations:** Institute of Physiological Chemistry, University of Ulm, 89081 Ulm, Germany; nadine.sperb@uni-ulm.de (N.S.); miltiadis.tsesmelis@uni-ulm.de (M.T.)

**Keywords:** PDAC, CAF, immune microenvironment, tumor microenvironment, cancer metabolism, CAF heterogeneity

## Abstract

Pancreatic ductal adenocarcinoma (PDAC) remains a lethal cancer. The poor prognosis calls for a more detailed understanding of disease biology in order to pave the way for the development of effective therapies. Typically, the pancreatic tumor is composed of a minority of malignant cells within an excessive tumor microenvironment (TME) consisting of extracellular matrix (ECM), fibroblasts, immune cells, and endothelial cells. Research conducted in recent years has particularly focused on cancer-associated fibroblasts (CAFs) which represent the most prominent cellular component of the desmoplastic stroma. Here, we review the complex crosstalk between CAFs, tumor cells, and other components of the TME, and illustrate how these interactions drive disease progression. We also discuss the emerging field of CAF heterogeneity, their tumor-supportive versus tumor-suppressive capacity, and the consequences for designing stroma-targeted therapies in the future.

## 1. Cancer-Associated Fibroblasts Are Key Players in PDAC

Pancreatic ductal adenocarcinoma (PDAC) is one of the most lethal solid cancers, with a 5-year relative survival rate of 9% [1]. Currently representing the fourth leading cause of cancer-related deaths in the United States, pancreatic cancer is predicted to become the second most lethal cancer type by 2030 [1,2]. Despite extensive research efforts over the past decades, progress in the diagnosis and treatment of the disease remains elusive. The majority of patients are diagnosed at an advanced stage when the tumor is unresectable and metastasis is already present.

Large-scale cancer genomic studies have revealed a complex mutational landscape in PDAC. A total of 32 recurrently mutated genes have been identified that aggregate into 10 molecular mechanisms and primarily drive the initiation and progression of the disease [3]. The most frequent oncogenic events include activating mutations of *KRAS* in over 90% of cases as well as mutations in *TP53*, *CDKN2A*, and *SMAD4* in over 50%, among a milieu of diverse genes mutated at low prevalence [4].

Fibrotic desmoplasia is one of the hallmarks of PDAC development, while cancer cells represent only a minority of the tissue mass in a pancreatic tumor. It is clear now that the dense fibrotic stroma is not just a bystander, but an active player during PDAC progression. This desmoplastic reaction, which may exceed 90% of the entire tumor volume, is characterized by the recruitment and activation of cancer-associated fibroblasts (CAFs), extensive extracellular matrix (ECM) deposition and remodeling, changes in immune surveillance, and altered vasculature. Multiple studies demonstrated that the stromal response and consequently the altered interactions between cancer cells and their surrounding environment promote tumor progression, invasion, metastasis, and chemoresistance [5,6,7,8].

**Figure 1:**
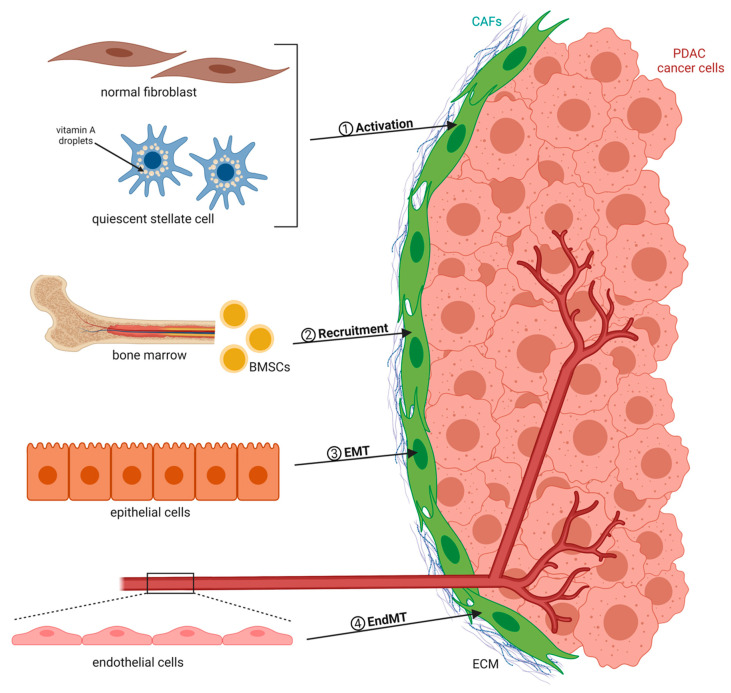
Heterogenous sources and activation mechanisms of cancer-associated fibroblasts (CAFs). CAFs can originate from pre-existing fibroblasts and quiescent stellate cells (via activation), bone marrow-derived mesenchymal stem cells (BMSCs) (via recruitment), epithelial cells (through epithelial-to-mesenchymal transition (EMT)), as well as from endothelial cells (through endothelial-to-mesenchymal transition (EndMT)). Abbreviations: pancreatic ductal adenocarcinoma (PDAC); extracellular matrix (ECM).

CAFs are recognized as the key cell population and active component of the tumor microenvironment (TME), which undergo morphological and functional changes when compared to normal fibroblasts. They secrete ECM proteins as well as soluble factors such as chemokines and cytokines [9,10]. There exist numerous sources of CAFs that group into four major categories (Figure 1). CAFs can originate from quiescent resident fibroblasts that are reprogrammed by adjacent tumor cells to form protumorigenic CAFs. For example, cancer cell-derived transforming growth factor-β (TGF-β) represents one of the most important and well-studied factors inducing CAF activation [11,12]. Interleukin-6 (IL-6), Sonic hedgehog (SHH), and platelet-derived growth factor (PDGF) are also known fibroblast-activating factors that stimulate the production of CAFs, with the latter being an essential initiator of the desmoplastic reaction in tumors [13,14,15,16]. In addition to extracellular molecules such as growth factors and cytokines, other mechanisms of CAF activation have been reported. Exosomes released by cancer cells can transfer protein, RNA, and microRNA (miRNA) to stromal fibroblasts, thereby contributing to their recruitment and activation [17,18]. The education process can be further mediated by local hypoxia and reactive oxygen species (ROS). As an example, ROS were found to promote the conversion of fibroblasts into myofibroblasts through the accumulation of the hypoxia-inducible factor (HIF)-1α transcription factor, whereas antioxidants were shown to reduce HIF-1α levels, thus inhibiting numerous myofibroblast features [19]. Moreover, widespread epigenetic reprogramming has recently emerged as a new mechanism driving de novo differentiation into CAFs [20]. Besides resident tissue fibroblasts, pancreatic stellate cells (PSCs) represent a distinct cell type and are categorized as CAFs when seen in cancer tissue. Once activated, these cells exhibit a loss of vitamin A reserves and acquire a myofibroblast-like phenotype, similar to activated normal fibroblasts. PSCs are generally considered to be the most important source of CAFs in the PDAC context and are responsible for the majority of the desmoplastic reaction [21,22]. In addition to the local CAF precursors, increasing evidence suggests that bone marrow-derived mesenchymal stem cells MSCs (BMSCs) are actively recruited to the tumor site, where they can differentiate into a substantial proportion of CAFs [23,24]. The third and fourth sources of CAFs are epithelial or endothelial cells that are in close proximity to cancer cells and undergo epithelial-to-mesenchymal transition (EMT) or endothelial-to-mesenchymal transition (EndMT), respectively [25,26].

Several cellular markers allow discriminating between quiescent fibroblasts and activated CAFs. The most commonly used CAF biomarkers to date comprise α-smooth muscle actin (α-SMA), fibroblast activation protein (FAP), fibroblast-specific protein 1 (FSP1), platelet-derived growth factor receptor-α (PDGFRα), PDGFRβ, and podoplanin (PDPN/gp38) [27,28,29]. α-SMA was initially considered as “the” pan-CAF marker, as, specifically, PSCs upon activation lose their vitamin A lipid droplet expression and start to exhibit myofibroblast-like characteristics staining positive for α-SMA [21]. Analysis of CAFs in a genetically engineered PDAC mouse model confirmed that approximately 75% of PDAC CAFs indeed express α-SMA. However, using a second marker, PDGFRα, the authors observed only limited overlap with the α-SMA positive fraction and an additional population (~16%) that expressed neither of the two markers [30]. In line with this, another study identified basically two separate CAF populations, being either α-SMA or Fsp1 positive [28]. Together, this indicates that there exists no unique CAF biomarker to define the entire CAF population. Instead, markers to identify CAFs have demonstrated heterogeneity in expression, pointing towards the existence of distinct subpopulations, where each subset is characterized by a specific combination of several markers. This heterogeneity is important to consider when comparing studies and interpreting results that utilized different markers or panels to either isolate or target CAFs.

## 2. Bidirectional Cancer Cell–CAF Crosstalk Promotes Tumor Progression

**Figure 2:**
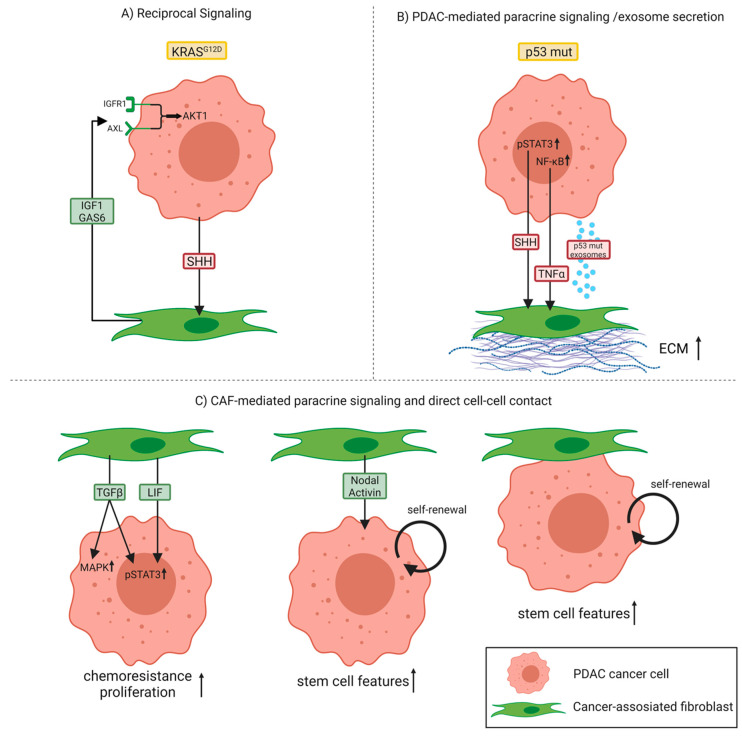
Different mechanisms of crosstalk between pancreatic ductal adenocarcinoma (PDAC) cancer cells and cancer-associated fibroblasts (CAFs). (**A**) Communication between PDAC cancer cells harboring mutant KRAS^G12D^ and CAFs can occur via a reciprocal signaling network. (**B**) PDAC cancer cells with mutant p53 (p53 mut), which exhibit persistent activation of signal transducer and activator of transcription 3 (STAT3) and enhanced activity of the nuclear factor-κB (NF-κB) signaling pathway, influence CAFs in a paracrine manner or through exosome secretion, stimulating the deposition of extracellular matrix (ECM). (**C**) CAFs can also secrete paracrine factors acting on cancer cells that lead to an upregulation of mitogen-activated protein kinase (MAPK) and STAT3 signaling and finally to PDAC progression and chemoresistance. Both CAF-secreted factors and direct PDAC–CAF interactions can further induce PDAC stem cell features, thereby promoting self-renewal capacity and invasiveness. Abbreviations: sonic hedgehog (SHH); insulin-like growth factor 1 (IGF1); insulin-like growth factor 1 receptor (IGFR1); growth arrest-specific gene 6 (GAS6); tumor necrosis factor-alpha (TNF-α); transforming growth factor-beta (TGF-β); leukemia inhibitory factor (LIF).

In 2008, Vonlaufen et al. demonstrated for the first time the tumor-promoting interactions between cancer cells and the stromal component in a physiologically representative in vivo situation [31]. Orthotopic co-injection of pancreatic cancer cells with PSCs into the tail of the pancreas of nude mice significantly enhanced the pancreatic tumor growth rate as well as the occurrence of regional and distant metastasis compared to the injection of cancer cells alone. Furthermore, they provided evidence that the interaction between tumor and stromal cells is not unidirectional (cancer cells influencing PSCs), but rather bidirectional because PSCs were found to significantly influence cancer cell survival through increasing proliferation and, at the same time, inhibiting apoptosis of the latter [31]. This bidirectional crosstalk or—to be more precise—the existence of a reciprocal signaling network between PDAC cells and CAFs/PSCs, was further confirmed using an in-depth phosphoproteomic approach [32]. This study revealed that pancreatic epithelial cells harboring mutant KRAS^G12D^, which represents the primary oncogenic driver in PDAC, engage pancreatic PSCs to subsequently instigate reciprocal signaling in the tumor cells (Figure 2A). Specifically, tumor cells with mutant KRAS^G12D^ communicate with PSCs via SHH signaling, which is transduced by PSCs, but not by KRAS^G12D^ PDAC cells. As a result, this allows KRAS^G12D^ PDAC cells to signal to PSCs via SHH, while at the same time remaining insensitive to autocrine SHH. These stimulated PSCs react by increasing stromal production of growth factors like insulin-like growth factor 1 (IGF1) and growth arrest-specific gene 6 (GAS6), which in turn affect the phosphoproteome of KRAS-mutant tumor cells. Consequently, PSC-mediated reciprocal signaling regulates tumor cell proliferation, protects cancer cells from apoptosis, and increases mitochondrial capacity via an IGF1R/AXL-AKT axis.

A recent study indicated that the specific genotype of tumor cells can directly influence CAF function. Particularly, differences in the p53 status of PDAC cells were shown to affect both the local and long-range paracrine signaling to surrounding CAFs [29]. Here, they used the poorly metastatic Pdx1-Cre;LSL-Kras^G12D/+^;p53^flox/+^ (KPflC) mouse model—which the authors call p53 null although genetically these mice are heterozygous—and the highly metastatic Pdx1-Cre;LSL-Kras^G12D/+^;LSL-p53^R172H/+^ (KPC) mouse model harboring a gain-of-function mutant p53 (p53 mutant). Comparative analysis of primary tumors of both groups revealed that p53 mutant PDAC cells can educate CAFs to establish a prometastatic and chemoresistant microenvironment. These p53 mutant PDAC cells exhibit enhanced activity of the nuclear factor-κB (NF-κB) signaling pathway compared to p53 null cancer cells and secrete higher levels of the NF-κB target gene tumor necrosis factor-α (TNF-α). TNF-α stimulates the expression and deposition of stromal perlecan by CAFs, an ECM component that contributes to an environment permissive to invasion and metastasis (Figure 2B). Remarkably, these p53 mutant-educated CAFs were shown to induce invasion of, normally, poorly invasive p53 null cancer cells to a similar extent as the highly invasive p53 mutant cancer cells, indicating that aggressive phenotypes can be transferred to less aggressive cells across the tumor. In addition, they could demonstrate that CAFs educated by p53 null cancer cells can be reprogrammed by interacting with either p53 mutant cancer cells or their CAFs leading to the acquisition of more invasive and metastatic features, thus behaving like p53 mutant-educated CAFs. To generalize, as cancer cells of distinct genotypes can reside in the same tumor, the genetic aberrations within PDAC cells in combination with the extensive plasticity exhibited by CAFs may represent important drivers of CAF heterogeneity.

Furthermore, phenotypically aggressive cancer cells are also able to confer protumorigenic characteristics through exosomal factors to both other tumor cells and fibroblasts. Novo et al. reported that p53 mutant cancer cells produce exosomes that activate Rab-coupling protein (RCP)-dependent integrin recycling in p53 null recipient cells to evoke migratory characteristics associated with p53 mutant’s invasive gain-of-function [33,34]. They identified decreased expression of podocalyxin (PODXL), a highly sialylated glycoprotein, in p53 mutant exosomes as a factor driving this process upon intercellular transfer. Second, exosomes from p53 mutant cancer cells influenced integrin trafficking in normal fibroblasts as well, leading to increased deposition and altered ECM architecture [34] (Figure 2B). When fibroblasts were treated with exosomes derived from p53 mutant cancer cells, they produced an ECM with a similar stiffness compared to fibroblasts exposed to exosomes from p53 null cancer cells. However, the adhesive properties of the ECM differed between the two conditions, with ECM generated by fibroblasts treated with p53 mutant exosomes being less sticky. This resulted in less well-established cancer cell–ECM contact structures, thereby facilitating tumor cell migration and invasion. Moreover, they observed alterations to ECM organization not only in the primary tumor, but also in the lung of animals possessing p53 mutant-driven PDAC, suggesting that mutant p53 can modify the microenvironment even in distant organs in a way to support invasive growth.

From a molecular perspective, mutated or biallelic loss of p53 was further found to contribute to persistent activation of Janus kinase (JAK)/ signal transducer and activator of transcription 3 (STAT3) signaling, which acts as an important regulator of stromal remodeling in both murine and human PDAC. More precisely, persistent STAT3 activation mediates SHH pathway activation in stromal cells, which subsequently leads to an enhanced desmoplastic stromal response through paracrine stimulation of CAFs/PSCs, increased tumor growth, and resistance to gemcitabine, the chemotherapeutic standard of care for PDAC [35] (Figure 2B). In line with this, PDAC patients whose tumors exhibited lower levels of phosphorylated STAT3 and functional p53 had a significantly prolonged overall survival compared to patients with high levels of phosphorylated STAT3 and p53 mutation, emphasizing the relevance of this p53-controlled JAK/STAT3-dependent mechanism.

Apart from the p53 status, impairment of TGF-β is another example of how the PDAC genotype dictates the extent and characteristics of the fibrotic response. TGF-β signaling plays an important role in PDAC progression, as indicated by the fact that Smad4, a well-known TGF-β downstream effector, is inactivated in over 50% of PDAC patients and the *type II TGF-β receptor (Tgfbr2)* gene is altered in a smaller subset of human PDAC [36]. Using patient samples and mouse models of pancreatic cancer, Laklai and colleagues investigated the architecture and mechanics of collagen fibers adjacent to epithelial lesions [37]. They demonstrated that especially PDACs with impaired TGF-β signaling have elevated epithelial STAT3 activity and show a unique, highly rigid, matricellular-stromal phenotype. Those PDAC genotypes activating JAK/STAT3 signaling were found to promote epithelial contraction via integrin and Yes-associated protein 1 (YAP1) mechanosignaling and led to the reorganization of the adjacent ECM into thick bundles. The resulting stiffer, matricellular-enriched fibrosis promoted tumor progression. In contrast, epithelial Stat3 ablation prolonged survival of Ptf1α-Cre;LSL-Kras^G12D/+^;Tgfbr2^flox/flox^ mice by reducing stromal stiffening and epithelial contractility induced by the loss of TGF-β signaling, indicating that the composition and mechanics, rather than just bulk ECM abundance, may serve as an indicator for PDAC aggressiveness. Together, this suggests that normalizing the biomechanical properties of these tumors may represent a novel strategy to treat PDAC.

In addition to the well-established activation of STAT3 in epithelial cells during PDAC progression [38], tumor cells were also shown to activate this pathway in CAFs as a mechanism to support PDAC cell growth [39]. Specifically, upon direct cell–cell contact, PDAC cells induced suppressor of cytokine signaling 1 (*SOCS1)* gene methylation and downregulation in CAFs, which normally functions as a STAT inhibitor. This resulted in phosphorylation of STAT3 followed by the secretion of protumorigenic cytokines and growth factors such as IGF1 to facilitate malignant growth and progression. Additionally, the functional relevance of this interaction was confirmed in vivo, demonstrating that patient-derived CAFs with epigenetic silencing of *SOCS1* promoted stronger growth of PDAC xenografts in mice than CAFs without *SOCS1* methylation [39].

Furthermore, CAFs per se can also serve as a source of paracrine factors acting on cancer cells to activate STAT3 [40]. Systematic proteomic investigation of secreted disease mediators identified the leukemia inhibitory factor (LIF) as a key paracrine factor from CAFs for STAT3 activation in cancer cells of the epithelial compartment. Both pharmacologic blockade of the LIF receptor and genetic *Lifr* deletion in pancreatic epithelial cells significantly slowed down tumor progression and improved chemotherapy efficacy to increase survival in PDAC mouse models, highlighting a critical role for stroma-derived LIF in PDAC progression as well as chemoresistance (Figure 2C). In line with this, another study reported an upregulation of STAT3 and mitogen-activated protein kinase (MAPK) signaling in a subpopulation of highly proliferative and invasive PDAC cancer cells upon co-culture of patient-derived PDAC cells with CAFs [41]. CAF-secreted TGF-β was identified to be responsible for these phenotypic changes. Using a neutralizing antibody against TGF-β abrogated the pro-proliferative effects in PDAC cell lines cultured in CAF-conditioned medium, confirming the mechanistic role of TGF-β in PDAC: CAF crosstalk (Figure 2C). Besides TGF-β, other members of the TGF-β family, namely, CAF-secreted Nodal and Activin, were established as relevant factors in tumor-stroma crosstalk with the capacity to enhance stemness in adjacent cancer cells [42]. According to the authors, Nodal/Activin is not only produced and secreted by pancreatic cancer stem cells in an autocrine fashion, but also by CAFs, thereby promoting the self-renewal capacity and invasiveness of primary pancreatic cancer stem cells. In contrast to the proposed paracrine mechanism, a recent study suggests that direct PDAC–CAF interactions promoted PDAC stem cell features by signaling through β1-integrin and activating focal adhesion kinase (FAK), confirming the multiple layers and possibilities of communication between the two compartments in driving progression of the disease [43] (Figure 2C).

## 3. Metabolic Reprograming Mediated by CAFs

Altered metabolism is one of the hallmarks of cancer cells. Over decades, especially the Warburg effect, describing a metabolic shift in cellular energy production from mitochondrial oxidative phosphorylation to increased oxygen-independent glycolysis, even in the presence of sufficient oxygen, has been established as common knowledge [44]. However, recently, a new paradigm of cancer metabolism has emerged, named the “reverse Warburg effect”, wherein tumor cells and CAFs become metabolically coupled [45]. In this model, cancer cells literally behave as “metabolic parasites”, as they use oxidative stress via secreted hydrogen peroxide or miRNAs to extract nutrients from adjacent stromal cells to cope with the otherwise nutrient-poor environment [46,47]. More precisely, this targeted oxidative stress in CAFs triggers the activation of NF-κB and HIF-1α, leading to the onset of inflammation, autophagy, mitophagy, and aerobic glycolysis in the TME. As a consequence, CAFs start to produce energy-rich metabolites (lactate, ketones, and glutamine) and chemical building blocks (nucleotides, amino acids, and fatty acids) that upon transfer fuel mitochondrial biogenesis and oxidative metabolism in cancer cells [48,49].

Sherman et al. provided evidence for this growth-promoting metabolic crosstalk between stromal CAFs and epithelial cancer cells by exposing several PDAC cell lines to cell-free extracts of secreted factors from cultured stromal cells [50]. This treatment was sufficient to dramatically increase the survival of cultured PDAC cell lines under relevant conditions of nutrient challenge. They confirmed that the survival response was linked to a rapid change in transcriptional networks, driving core metabolic pathways in the tricarboxylic acid (TCA) cycle, anabolic metabolism, and cell growth. Interestingly, the transcriptional and metabolic changes induced by the stromal secretome overlapped with those induced by oncogenic KRAS, indicating that the stromal reaction might cooperate with oncogenic alterations in KRAS to drive pancreatic cancer progression [50]. Sousa and colleagues went one step further by identifying an individual CAF-secreted component with the capacity to drive metabolic reprogramming [51]. They found that cancer cells stimulated CAF autophagy, which eventually caused secretion of high levels of the amino acid alanine. This stromal alanine ultimately outcompeted glucose and glutamine-derived carbon in PDAC cells to fuel the TCA, and thus non-essential amino acids and lipid biosynthesis to aid tumor cell proliferation even in low-nutrient conditions [51]. Beyond the direct supply of amino acids to PDAC cells, CAFs can also indirectly serve as a source of amino acids by producing a collagen-rich ECM. It was shown that especially extracellular collagen represents a proline-rich reservoir, which can be taken up by PDAC cells and used as a nutrient pool when other fuels are limited, thus highlighting the metabolic flexibility of pancreatic cancer [52]. In line with this, PDAC cells can scavenge and consume extracellular proteins via macropinocytosis [53]. Using a microdevice to deliver labeled extracellular proteins into tumors of KRAS-driven PDAC mice, albumin and fibronectin uptake and catabolic degradation were observed exclusively by cancer cells.

Apart from amino acids, stroma-derived lipids were also shown to support PDAC metabolism and growth. Generally, CAFs are characterized by a pronounced lipid metabolic shift upon activation, including remodeling of the intracellular lipidome and secretion of abundant lipids. Following uptake by PDAC cells, these CAF-derived lipids are channeled to various lipid pools, including phospholipids for membrane synthesis and growth [54]. Besides contributing to biomass production, particularly CAF-secreted lysophosphatidylcholine (LPC) was further identified as a potent precursor for signaling lipids in PDAC cells, thus promoting PDAC cell proliferation, migration, and AKT activation [54].

Finally, the translocation of metabolic substrates from CAFs to tumor cells can occur via exosomes as well. Another metabolic study convincingly demonstrated by isotope tracing that CAF-derived exosomes supply intact metabolites such as amino acids, lipids, and TCA cycle intermediates directly to cancer cells, which utilize them for maintaining proliferation under nutrient-deprived conditions [55]. Interestingly, in addition to providing metabolic support to cancer cells, CAF-derived exosomes were also found to play an active role in regulating chemoresistance of cancer cells. CAFs exposed to gemcitabine significantly increased the release of exosomes, displaying higher amounts of Snail mRNA which encodes a transcription factor mediating chemoresistance, EMT, and metastasis [56]. Consequently, this hypersecretion of Snail mRNA resulted in increased cell proliferation and survival of recipient cancer cells following exosomal uptake, which could be reverted by using an inhibitor of exosome release. However, CAF-mediated exosomal transfer is not the only mechanism to facilitate PDAC cell proliferation in the context of chemotherapy treatment. Another group recently identified CAF-secreted deoxycytidine as a factor protecting PDAC cells from gemcitabine toxicity [57]. Mechanistically, deoxycytidine was shown to inhibit the intracellular processing of the drug in PDAC cells likely through the competition for the deoxycytidine kinase, thereby reducing the effect of gemcitabine and other nucleoside analogs on cancer cells. More recently, lactate import via the lactate importer monocarboxylate transporter-1 (MCT1) into PDAC cells was reported to exert a protective effect against gemcitabine-induced apoptosis [58]. In contrast to MCT1-expressing PDAC cells, MCT1-negative PDAC cells did not exhibit alterations in gemcitabine-induced apoptosis rates when receiving lactate pretreatment, strengthening the MCT1 dependency of this lactate-mediated effect. Notably, analysis of tumor tissue from PDAC patients revealed strong expression of the lactate exporter MCT4 in the desmoplastic stroma, thus implying lactate shuttling between the two compartments. Moreover, MCT1-driven lactate import in PDAC cells under glucose starvation mimicking low nutrient conditions primed PDAC cells for a chemoresistant phenotype and favored stemness properties after their re-exposure to glucose compared to PDAC cells without lactate pretreatment. This study highlights that the MCT1-mediated import of lactate in PDAC cells not only confers an energy-rich metabolite to the cells, but is also an efficient driver of metabostemness associated with resistance to therapy [58].

## 4. CAFs Modulate the Immune Microenvironment and Crosstalk with Additional Cell Types in the Desmoplastic Stroma

It is widely accepted that in many types of cancer immune cells do not only exhibit tumor-suppressive functions, but also promote the immunosuppressive TME and lead to tumor growth, invasion, and metastasis. PDAC, one of the most immunosuppressive tumors, educates resident and infiltrating immune cells towards this immunosuppressive state [59]. The immune cell compartment of the pancreatic TME mainly consists of tumor-associated macrophages (TAMs), myeloid-derived suppressor cells (MDSCs), regulatory T cells (Tregs), and few activated cytotoxic effector T cells (CD8^+^) [60].

Recent studies suggest CAFs as central players in the immune regulation of PDAC by different mechanisms, such as cytokine and chemokine secretion, antigen presentation, and immune cell adhesion [61]. Ene-Obong et al. investigated the distribution of different types of immune cells in the distinct stromal compartments of PDAC [62]. Analysis of PDAC tumors from human patients revealed that CD8^+^ T cells, Tregs (FoxP3^+^), B cells (CD20^+^), and natural killer (NK) cells (CD56^+^) could not penetrate the juxtatumoral stromal compartment (within 100 μm of tumor cells) efficiently, as the higher percentage was detected in the pan-stromal compartment (the rest of the tumor). Furthermore, knock-down of C-X-C motif chemokine ligand 12 (CXCL12), a chemoattractant of T cells, in primary PDAC-patient CAFs reduced the migration of PDAC-patient CD4^+^ and CD8^+^ T cells towards CAFs, showcasing the importance of the CXCL12/ C-X-C chemokine receptor 4 (CXCR4) axis in T cell trafficking [63]. Surprisingly, the majority of the macrophages (CD68^+^ cells) were detected to the juxtatumoral stroma. Within the stroma, collagen can be cleaved by fibroblast activation protein (FAP) and therefore act as a substrate for macrophage binding [64]. Inhibition of FAP in mice injected with the PDAC cell line Panc02 reduced the total number of the macrophages infiltrating the stroma, highlighting the importance of FAP-expressing CAFs for the macrophage abundance in the tumor stroma [65].

TAMs can exert either beneficial or detrimental effects in tumor growth and metastasis, depending on their polarization (M1 or M2-like macrophages) [66]. Zhang et al. investigated the role of CAFs in macrophage polarization towards the M2 immunosuppressive state [67]. Stimulation of human peripheral blood mononuclear cells (PBMCs) with pancreatic CAF-conditioned medium enhanced the expression of M2 markers CD206 and CD163. Analysis of the CAFs secretome identified macrophage colony-stimulating factor (M-CSF) as a potential regulator of this response. Indeed, inhibition of M-CSF partially abrogated the induction of the M2 polarization. An additional pathway promoting the M2 polarization by stromal factors was identified by Andersson and colleagues using Panc02-injected mice [68]. Specifically, CAFs secreted IL-33, which bound to the suppression of tumorigenicity 2 (ST2) receptor of TAMs and significantly upregulated the expression of M2-related genes. Further analysis revealed that ST2 receptor activation resulted in an NF-κB-dependent matrix metallopeptidase 9 (MMP9) expression in TAMs. Knock-out of IL-33 in Panc02-injected mice reduced the proportion of mice with visible metastatic nodules to the lungs by approximately 50%, highlighting the importance of IL-33/NF-κB/MMP9 axis in tumor metastasis.

MDSCs are a heterogeneous group of immature myeloid cells that originate from the bone marrow and are divided into two main categories: the granulocytic MDSCs, which morphologically resemble the neutrophils, and the monocytic MDSCs, which morphologically resemble the monocytes. MDSCs suppress the activity of T cells and NK cells and have been linked with immune-suppressive responses in various pathological conditions, and especially in cancer [69]. Mace and colleagues examined the role of pancreatic CAFs in MDSC differentiation [70]. Supernatant from human-derived pancreatic CAF cell lines contained MDSC-promoting cytokines (IL-6, vascular endothelial growth factor (VEGF), and M-CSF) and MDSC-attracting chemokines (monocyte chemoattractant protein-1 (MCP-1) and CXCL12). Incubation of PBMCs with CAFs supernatant promoted their differentiation to MDSCs (CD11b^+^ CD33^+^), while co-culture of T cells with the abovementioned differentiated MDSCs dramatically reduced the proliferation rate of T cells. Inhibition of IL-6 through a neutralizing antibody resulted in major inhibition of STAT3 phosphorylation in PBMCs and blocked their differentiation towards MDSCs. These data suggest that CAFs play an important role in the differentiation of MDSCs and the regulation of the immunosuppressive TME.

In a recent study, a CAF subpopulation with antigen presentation features (apCAFs) was detected in PDAC tumors from Pdx1-Cre;LSL-Kras^G12D/+^;LSL-p53^R172H/+^ (KPC) mice using single-cell RNA sequencing [71]. These apCAFs expressed genes belonging to the Major Histocompatibility Complex (MHC) class II family, which are mainly expressed by antigen-presenting cells (APCs). Co-culture of OTII-derived CD4^+^ T cells (specifically recognizing the ovalbumin (OVA) peptide) with OVA-loaded apCAFs or OVA-loaded APCs for 17 hours challenged the capacity of apCAFs to activate T cells [72]. OVA-loaded apCAFs indeed promoted the expression of the activation markers CD25 and CD69 by CD4^+^ T cells, although to a lower extent than OVA-loaded APCs. Immunohistochemistry (IHC) co-staining of human PDAC samples with the fibroblast marker PDGFRβ and MHC class II molecules verified the presence of apCAFs in human PDAC too [71].

In many solid tumors, increased CD8^+^ T cell infiltration is associated with a good prognosis [73]. Unfortunately, cancer and immune cells express checkpoint ligands such as programmed death-ligand 1 (PD-L1) and CTL-associated antigen 4 (CTLA-4), which bind to CD8^+^ T cells and repress their T-cell receptor (TCR) signaling, proliferation, and motility [62,74,75]. Treatment with checkpoint inhibitors such as anti-PD-L1 or anti-CTLA-4 antibodies can block the immune checkpoint signaling pathways and boost the immune response against tumor cells in many types of cancer [76].

Despite the advance in the field of cancer immunotherapy and the treatment of various forms of cancers with checkpoint inhibitors, immunotherapeutic treatment rarely displays a positive outcome in PDAC patients [77]. In most PDAC cases, the number of CD8^+^ T cells that can infiltrate the stroma and reach the tumor cells is low, which leads to significant low immunogenicity in this type of cancer [62,78]. Interestingly, PDAC is characterized by a vast desmoplastic reaction, which accounts for up to 90% of the tumor mass. The main cell populations residing in the stroma consist of immunosuppressive CAFs, MDSCs, and macrophages, while CD8^+^ T cells are relatively sparse [62,79]. Furthermore, it has been recently shown that extratumoral macrophages block CD8^+^ T cells from infiltrating the TME [80]. Additionally, another study has shown that FAP^+^ CAFs secrete CXCL12, which coat the tumor cells and block the accumulation of CD8^+^ T cells to the proximity of the tumor [81]. Thus, checkpoint inhibitors that could prevent CD8^+^ T cells from being inactivated have almost no effect on PDAC, as the CD8^+^ T cells cannot even infiltrate efficiently the stroma and reach the tumor cells. Combination therapies that promote the infiltration of the CD8^+^ T cells to the proximity of the tumor and, at the same time, protect them from getting inactivated could potentially display a positive outcome against PDAC. As described by Feig et al., combination treatment with depletion of FAP^+^ CAFs and administration of α-CTLA-4 or α-PD-L1 diminished the tumor growth by 15% after 6 days of treatment in KPC mice [81].

Recently, the role of CAFs in the immunosuppression of CD8^+^ T cells has been studied in PDAC. They observed that transforming growth factor β-induced (TGFBI), an extracellular matrix protein that is detected in PDAC, is mainly produced by CAFs within the stromal compartment of Pdx1-Cre;LSL-Kras^G12D/+^ (KC) mice [73,82]. Treatment of OTI (MHC class I-restricted OVA specific T cell receptor) cells with CAF-conditioned medium significantly reduced the proliferation rate of OTI cells, and the addition of anti-TGFBI-depleting antibody reversed the effect [73,83]. Thorough analysis identified that TGFBI interacts with CD61 on the surface of CD8^+^ T cells. Treatment of CD8^+^ T cells with TGFBI induced the internalization of CD61 and the phosphorylation of the lymphocyte-specific protein tyrosine kinase (Lck) at tyrosine residue 505 (Y505), subsequently inhibiting the TCR signaling pathway [73,84]. Interestingly, macrophages also expressed CD61, and binding of TGFBI to macrophages diminished the production of interferon-γ (IFN-γ) and TNF-α. More importantly, treating KC cell line-injected C57BL/6 mice with anti-TGFBI-depleting antibody significantly reduced the tumor volume and led to the accumulation of CD8^+^ T cells to the primary tumor. These CD8^+^ T cells were also characterized by increased expression of granzyme B, IFN-γ, TNF-α, and a significant reduction of the exhaustion marker programmed death 1 (PD-1). These results highlight the importance of CAFs and TGFBI in the immunosuppression of CD8^+^ T cells. All things considered, pancreatic CAFs, by regulating the activity and the attraction of immune cells, promote the formation of the immunosuppressive microenvironment in PDAC.

CAFs do not only crosstalk with cancer and immune cells, but can also interact with additional cells types in the desmoplastic stroma such as endothelial cells and neurons. Interestingly, CAFs promote angiogenesis in PDAC while, at the same time, they exhibit antiangiogenic features. CAFs secrete VEGF, angiopoietin-1, and hepatocyte growth factor (HGF), which increase the proliferation rate of endothelial cells and subsequently support angiogenesis [6,85,86]. Furthermore, co-culture of fibroblasts with metastatic pancreatic cancer cells stimulates the proliferation of fibroblasts and promotes secretion of the proangiogenic proteins CXCL8 and C-C motif ligand 2 (CCL2) [87]. On the other hand, CAFs express vasohibin-1, as well as stimulate pancreatic cancer cells to produce endostatin, both of which act as antiangiogenic factors [85,88,89].

A common adverse feature of pancreatic cancer is neural invasion (NI) [90]. NI is the pathological process in which cancer cells invade through the nerve or its surroundings and is generally associated with a poor prognosis [90,91]. Studies in patients have revealed that tumors with high nerve growth factor (NGF) expression levels exhibit more frequent NI [92]. Interestingly, there is evidence indicating a connection between CAFs and NI. In vitro experiments revealed that CAFs secrete HGF, which subsequently increases the expression of NGF in cancer cells. In the presence of CAF-conditioned media, pancreatic cancer cells were able to successfully migrate towards dorsal root ganglia (DRG), whereas knock-down of NGF or HGF significantly reduced the migration, highlighting the importance of CAFs in NI [91,93].

## 5. CAF Ablation Studies—Functional Evidence for a Tumor-Suppressive Role of CAFs

Based on the studies described above and others that strongly suggest a tumor-promoting function of CAFs in the context of PDAC, researchers have started working on approaches aiming at the ablation of CAFs in mouse models of pancreatic cancer. These efforts led to three seminal publications in 2014 that, despite using different systems, all surprisingly found that the depletion of CAFs worsened the outcome, resulting in poorly differentiated and more aggressive tumors with diminished animal survival [94,95,96].

Two of the studies depleted CAFs through genetic disruption or prolonged pharmacological inhibition of SHH, a ligand that stimulates CAFs [14,95,96]. Rhim et al. generated an Shh-knockout PDAC mouse model by crossing an Shh-floxed allele into the Pdx1-Cre;LSL-Kras^G12D/+^;p53^flox/+^;LSL-Rosa26^YFP/+^ (KPCY) model of pancreatic cancer, resulting in the loss of Shh-dependent stroma throughout tumor progression [95]. Contrary to the expectation that Shh loss would somehow impair tumorigenesis, these ShhKPCY mice developed tumors earlier and ultimately succumbed to the disease more rapidly and with a higher incidence of metastasis than their KPCY counterparts. As predicted, Shh-deficient tumors had reduced stromal content, but at the same time, such tumors were more aggressive and had undifferentiated histology with increased expression of EMT markers compared to well- to moderately differentiated KPCY tumors. Moreover, ShhKPCY tumors exhibited a substantial increase in blood vessel density accompanied by greater perfusion, which contributed to an increase in cancer cell proliferation, most likely due to the enhanced nutrient supply. Second, they also used a pharmacologic approach to inhibit canonical Hedgehog (Hh) signaling by targeting the essential pathway effector Smoothened (SMO). Normally, Hh signaling involves the secretion of SHH ligands by tumor cells, which subsequently bind to the Hh receptor Patched (PTCH) expressed by the stromal compartment. This in turn activates the Hh-transducing molecule SMO, a seven-transmembrane protein, and initiates the downstream signaling pathway cascade [97]. Pharmacologic inhibition of Hh signaling in the present study was achieved by treating Pdx1-Cre;LSL-Kras^G12D/+^;LSL-p53^R172H/+^ (KPC) mice with the SMO inhibitor IPI-926, starting from 8 weeks of age, a time point prior to PDAC formation, but in the presence of acinar-to-ductal metaplasia (ADM) and premalignant pancreatic intraepithelial (PanIN) lesions. Chronic SMO inhibition accelerated tumor growth, recapitulating the effect of genetic deletion of *Shh* in pancreatic tumors. Strikingly, nearly all of the IPI-926-treated mice had to be sacrificed following a period of rapid and severe weight loss. Although the authors could not fully explain the accelerated mortality, they hypothesized that stromal inhibition and associated changes in tumor–stroma crosstalk and consequently tumor metabolism may lead to increased cachexia, a characteristic wasting syndrome commonly seen in human PDAC patients. Consistent with the aforementioned findings, Lee and colleagues likewise observed acceleration of disease progression in three distinct PDAC mouse models by either genetic (pancreas-specific knock-out of *Shh*) or pharmacologic inhibition of Shh signaling [96]. They specifically focused on the balance between epithelial and stromal elements in response to acute pharmacological modulation of the pathway. Inhibition caused suppression of desmoplasia and accelerated the growth of epithelial elements, whereas activation using a small molecule agonist resulted in stromal hyperplasia and reduced growth of the PanIN epithelium. Thus, they concluded that the stromal response rather plays a restraining role during PDAC progression, reflecting the findings of several clinical trials with PDAC patients that had shown that therapeutic targeting of stromal fibrosis via Hh pathway inhibition in combination with cytotoxic chemotherapy added no benefit or was more harmful than chemotherapy alone [98].

Özdemir et al. used a different system, but obtained similar results upon CAF ablation in PDAC [94]. They crossed an α-SMA-thymidine kinase (α-SMA-tk) allele into the highly aggressive Ptf1a-Cre;LSL-Kras^G12D/+^;Tgfbr2^flox/flox^ (PKT) PDAC mouse model to temporarily deplete α-SMA-expressing proliferative cells, including CAFs, following systemic ganciclovir administration. In line with the studies described above, treatment with ganciclovir at either the noninvasive precursor (PanIN) or PDAC stage led to undifferentiated tumors and shortened survival with a reduced body weight at endpoint compared to mice without CAF-depleted tumors. Moreover, CAF depletion impacted the composition of the immune infiltrate in the TME, being particularly enriched in regulatory T cells (CD4^+^Foxp3^+^), which eventually resulted in the suppression of immune surveillance in tumors with reduced fibrosis. Finally, high stromal content also correlated with favorable outcomes in PDAC patients, implicating once more a protective effect of stroma and strengthening the need for caution in targeting CAFs in PDAC [94,99].

Collectively, these studies indicate that the desmoplastic stroma as a unilaterally protumorigenic niche calls for revaluation. There obviously exist certain CAF subtypes, some of which with protumorigenic features, whereas others—at least SHH-dependent, α-SMA-positive CAFs, or a subfraction of CAFs fulfilling these criteria—may have antitumorigenic properties restraining PDAC growth. In addition, as the function of the stroma is dynamic during disease progression and its cellular and noncellular components coevolve with the changes of the genetic landscape of cancer cells, also the timing of intervention matters [100].

## 6. The Emerging Field of CAF Heterogeneity

The divergent results of CAF manipulation in PDAC models clearly suggest the existence of intratumoral CAF heterogeneity, prompting a more detailed examination of this cell population. In 2017, Öhlund et al. succeeded in characterizing two spatially and functionally distinct CAF subtypes by utilizing a co-culture system of murine pancreatic organoids and PSCs [27]. They demonstrated that myofibroblastic CAFs (myCAFs) with elevated expression of α-SMA were most prevalent close to tumor foci and required juxtracrine interactions with cancer cells for their formation. In contrast, inflammatory CAFs (iCAFs) with low expression of α-SMA and high expression of inflammatory mediators, such as IL-6, IL-11, and LIF as well as the chemokines CXCL1 and CXCL2, were induced by secreted factors from cancer cells and were located more distantly from neoplastic cells within the dense stroma. Transcriptomic profiling further implied that myCAFs were contractile and stroma remodeling, while iCAFs were defined by a secretory phenotype, with the capacity to influence both cancer cells and other cell types present in the tumor in a paracrine manner. Importantly, these two subtypes could dynamically reverse from one cell state to the other, thus emphasizing the plasticity of CAFs that coexist in pancreatic cancer. In a later study, the same group sought to elucidate the mechanisms underlying the development of these CAF subtypes and identified IL-1 and TGF-β as tumor-secreted ligands that regulate CAF heterogeneity [101]. Specifically, IL-1 signaling through the IL-1 receptor (IL-1R) on CAFs was shown to promote the iCAF transcriptional program via NF-κB and subsequently mediate the induction of autocrine LIF, leading to the activation of JAK/STAT signaling. Conversely, inhibition of NF-κB activation impaired the ability of tumor organoid-conditioned media to induce inflammatory CAF marker genes, suggesting that that formation of iCAFs is dependent on NF-κB activation. TGF-β, on the other hand, antagonized IL-1-induced JAK/STAT signaling by downregulating IL-1R expression and shifted iCAFs to a myofibroblastic myCAF phenotype in vivo. These findings provide evidence that signaling gradients set up by cancer cells shape CAF heterogeneity in a way that proximity to tumor cells favors the myCAF phenotype through dominant TGF-β signaling, whereas CAFs that are more distantly to tumor cells rather experience IL-1/IL-1R signaling and thus acquire the iCAF state. Besides, a minor population of α-SMA/p-STAT double positive cells was seen as well, arguing for an additional subtype or an intermediate state between iCAF and myCAFs.

The presence of iCAFs and myCAFs was independently confirmed in human tissue specimens. Furthermore, it was demonstrated that stromal heterogeneity is evident even in premalignancy and throughout cancer progression in a stage-specific manner [102]. By analyzing precursor low-grade and high-grade intraductal papillary mucinous neoplasms (LGD- and HGD-IPMN) as well as PDAC, it was found that iCAFs solely associate with the PDAC state while being absent in the noninvasive dysplastic lesions. Interestingly, the emergence of iCAFs in the PDAC stage paralleled with a decrease in cytotoxic T cell and increase in myeloid-suppressive proportions, characteristic of an immunosuppressive microenvironment. On the contrary, the myCAF population was rarely observed in LGD-IPMNs, but highly represented in HGD-IPMNs, implying that activation of fibroblasts of the myCAFs phenotype is a rather early event already occurring in the noninvasive setting [102]. Likewise, analysis of low-passage patient-derived CAF primary cultures revealed the existence of similar subtypes resembling the iCAF and myCAF phenotypes, which indicates that the transcriptional heterogeneity can at least temporarily be maintained in the in vitro culture condition [22].

Most recently, a third CAF subtype was identified using single-cell RNA sequencing (scRNA-seq), which was termed “antigen-presenting CAF” (apCAF) [71]. ApCAFs express MHC class II and invariant chain CD74 and have the capacity to activate CD4^+^ T cells, suggesting an immunomodulatory role and adding more complexity to CAF heterogeneity. Additionally, apCAFs also expressed other unique markers such as serum amyloid A3 (SAA3), which was previously described as a key mediator of the protumorigenic activity of CAFs [30]. Under suitable culture conditions, apCAFs could convert into myCAFs, strengthening the hypothesis that CAF subpopulations represent interconvertible cell states, rather than endpoints in differentiation [27,71]. However, intratumoral signals that induce apCAF formation and activation have not been identified yet.

An independent scRNA-seq study demonstrated the existence of three distinct molecular subtypes of fibroblasts in the normal mouse pancreas (FB1, FB2, and FB3), which gave rise to two distinct CAF populations (FB1 and FB3) across different advanced-stage PDAC mouse models [103]. The FB1 transcriptional profile most closely represented the iCAF phenotype, while the FB3 population exhibited myofibroblastic properties together with the expression of some MHC class II associated genes, perhaps indicating a hybrid population consisting of myCAFs and apCAFs. This again supports the concept of intratumoural CAF heterogeneity in PDAC, albeit with slightly different clustering [103].

A more recent study described the single-cell landscape of CAFs in pancreatic cancer during in vivo tumor evolution in the Pdx1-Cre;LSL-Kras^G12D/+^;p16/p19^flox/flox^ (KPP) PDAC mouse model [104]. Two separate fibroblast lineages were characterized that coevolve during tumor progression driven by TGF-β and IL-1, consistent with previous findings [101]. With tumor progression, they specifically observed an increase in the frequency of CAFs programmed by TGF-β and expressing the leucine-rich repeat containing 15 (LRRC15) protein encoding a conserved transmembrane protein. These LRRC15^+^ CAFs, which clustered with myCAFs, surrounded tumor islets and were absent from normal pancreatic tissue. Notably, TGF-β-responsive LRRC15^+^ CAFs represented a prominent population in human PDAC samples, and data from recent clinical trials revealed that this signature correlated with a poorer response to immunotherapy, requiring further investigation of the functional relationship between this myofibroblastic CAF population and the antitumor immune response. Moreover, they also traced individual CAF populations back to their non-malignant ancestor and observed differences in the murine versus the human situation. Whereas pre-existing fibroblast heterogeneity in normal tissue dictated the developmental trajectories of murine CAFs, there was no baseline heterogeneity in the human non-malignant tissue fibroblasts. Rather non-malignant human fibroblasts were found to evolve towards a single early CAF which then gives rise to either a TGF-β- or IL-1-programmed CAF. However, as the analyzed human tissues were not truly normal, the authors could not exclude that non-malignant fibroblasts had already undergone changes that masked baseline heterogeneity [104].

Additionally, the cell of origin in general can act as another factor contributing to CAF heterogeneity. In this respect, Waghray and colleagues identified and characterized mesenchymal stem cells (MSCs) as a unique subpopulation of CAFs, which they designated as cancer-associated MSCs (CA-MSCs) [105]. Low-passage PDAC-derived CAF cultures contained between 1 and 20% CA-MSCs exhibiting MSC characteristics such as multipotent differentiation potential and the ability to form colonies. These CA-MSC exclusively secreted the cytokine granulocytic–macrophage colony-stimulating factor (GM-CSF), resulting in markedly enhanced growth, invasion, and metastatic potential of PDAC cancer cells, which express the respective GM-CSF receptor. Collectively, this implies a critical role for GM-CSF in mediating mesenchymal–epithelial crosstalk in PDAC. Besides facilitating cancer invasion, CA-MSCs were also found to regulate macrophage polarization in a tumor-promoting fashion [106,107].

**Figure 3:**
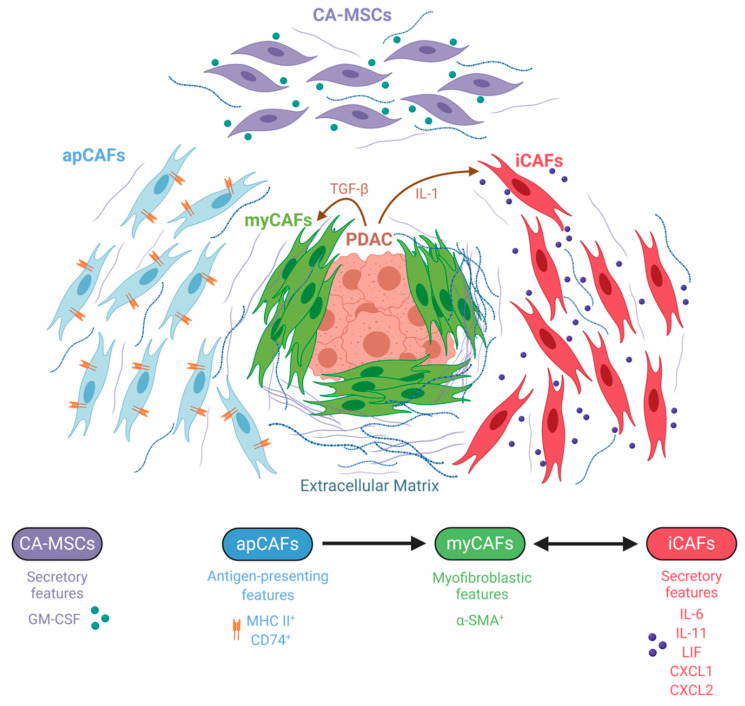
Schematic representation of the so far identified distinct cancer-associated fibroblast (CAF) subtypes in pancreatic ductal adenocarcinoma (PDAC). Three different CAF subsets have been described: (i) myofibroblastic CAFs (myCAFs), which proximally surround neoplastic cells and are defined by high alpha-smooth muscle actin (α-SMA) expression; (ii) inflammatory CAFs (iCAFs), which reside more distantly and are characterized by a secretory phenotype; and (iii) antigen-presenting CAFs (apCAFs) with immunomodulatory features. Literature suggests that CAFs are dynamic and can acquire different phenotypes. Transforming growth factor-beta (TGF-β) and interleukin-1 (IL-1) are tumor-secreted ligands that promote CAF heterogeneity with respect to the myCAF and iCAF phenotype, while the intratumoral trigger for apCAF formation remains unknown. Cancer-associated MSCs (CA-MSCs) represent another unique subtype with characteristics of mesenchymal stem cells (MSCs) and have a secretory phenotype influencing PDAC cancer cells. Most likely additional subtypes will be discovered in the future. Abbreviations: granulocytic-macrophage colony-stimulating factor (GM-CSF); major histocompatibility complex class II (MHC II); leukemia inhibitory factor (LIF); C-X-C motif chemokine ligand (CXCL).

Altogether, the field of fibroblast heterogeneity is still in its infancy. More subpopulations, except the myofibroblastic, inflammatory, antigen-presenting CAFs; CA-MSCs; and additional sub-classifications within the existing classes will likely be identified (Figure 3). For future work, it will be crucial to further deepen the knowledge about the dynamics, plasticity and origins of these heterogeneous populations during disease progression in order to be capable of designing rational stroma-targeted therapies.

## 7. Conclusions and Future Perspectives

CAFs are one of the most significant components in the TME where they can fulfill both protumorigenic as well as antitumorigenic functions depending on the stage of tumorigenesis, their spatial location, specific interaction partners, and the tumor genotype. Understanding this diversity, particularly with respect to distinct transcriptional programs driving the heterogeneous phenotypes, will hopefully facilitate the development of effective therapies for this disease.

Generally, several lines of evidence suggest that normalization or selective depletion of certain subsets rather than widespread ablation represents a more promising approach to stromal targeting in PDAC. Given the myCAF/iCAF theory, attempts to deplete CAFs based on their α-SMA expression may have preferentially eliminated tumor-restraining myCAFs while leaving other tumor-supportive CAF populations intact [27,94]. Considering that iCAFs secrete high levels of chemokines and cytokines that play a vital role in tumorigenesis and disease progression, it can be assumed that this CAF subfraction facilitates the aggressive tumor spread, although this association has yet to be explicitly proven. Successful conversion of iCAFs to the myCAF state would have at least two benefits: first, depletion of iCAFs would reduce the secretion of tumor-promoting cytokines and chemokines and, second, pushing iCAFs into a more myofibroblastic state would lead to a notable increase of the α-SMA-positive CAF population that has been previously shown to rather restrain disease progression [94]. However, it remains to be elucidated whether targeting a specific CAF subpopulation will have lasting effects, given their suggested capacity to dynamically reverse from one state to another. In addition, shifting the ratio towards a myofibroblastic state would also favor myCAF-derived desmoplasia which consequently might impede drug delivery, resulting in poorer responses to therapy [104].

Overall, due to the described complex interactions between CAFs, tumor cells, and other TME components, relevant in vivo models need to be established in the future that will allow the manipulation of individual CAF subtypes or their cellular precursors within the right tissue context. This will help us to obtain a more nuanced understanding of the function of this multifaceted cell population and to hopefully develop effective anti-CAF therapies for this devastating cancer.

## Abbreviations

ADM	Acinar-to-ductal metaplasia
apCAFs	Antigen-presenting CAFs
APCs	Antigen presenting cells
α-SMA	alpha-Smooth muscle actin
α-SMA-tk	alpha-Smooth muscle actin-thymidine kinase
CA-MSCs	Cancer-associated mesenchymal stem cells
CAFs	Cancer-associated fibroblasts
Cre	Cre recombinase
CTLA-4	Cytotoxic T-lymphocyte-associated protein 4
BMSCs	Bone marrow-derived mesenchymal stem cells
CCL2	C-C motif ligand 2
CDKN2A	Cyclin-dependent kinase inhibitor 2A
CXCL1	C-X-C motif chemokine ligand 1
CXCL2	C-X-C motif chemokine ligand 2
CXCL8	C-X-C motif chemokine ligand 8
CXCL12	C-X-C motif chemokine ligand 12
CXCR4	C-X-C chemokine receptor 4
DC	Dendritic cells
DRG	Dorsal root ganglia
ECM	Extracellular matrix
EndMT	Endothelial-to-mesenchymal transition
EMT	Epithelial-to-mesenchymal transition
FAK	Focal adhesion kinase
FAP	Fibroblast activation protein
FoxP3	Forkhead box P3
FSP1	Fibroblast-specific protein 1
GAS6	Growth arrest-specific gene 6
GM-CSF	Granulocytic-macrophage colony-stimulating factor
HGF	Hepatocyte growth factor
HH	Hedgehog
HIF-1α	Hypoxia-inducible factor 1-alpha
iCAFs	inflammatory CAFs
IFN-γ	Interferon-gamma
IGF1	Insulin-like growth factor 1
IGF1R	Insulin-like growth factor 1 receptor
IHC	Immunohistochemistry
IL-1	Interleukin-1
IL-1R	Interleukin-1 receptor
IL-6	Interleukin-6
IL-11	Interleukin-11
IL-33	Interleukin-33
JAK	Janus kinase
KC	Pdx1-Cre;LSL-Kras^G12D/+^
KPC	Pdx1-Cre;LSL-Kras^G12D/+^;LSL-p53^R172H^
KPCY	Pdx1-Cre;LSL-Kras^G12D/+^;p53^flox/+^;LSL-Rosa26^YFP/+^
KPflC	Pdx1-Cre;LSL-Kras^G12D/+^;p53^flox/+^
KPP	Pdx1-Cre;LSL-Kras^G12D/+^;p16/p19^flox/flox^
KRAS	Kirsten rat sarcoma virus
LCK	Lymphocyte-specific protein tyrosine kinase
LGD IPMN	Low-grade intraductal papillary mucinous neoplasm
HGD IPMN	High-grade intraductal papillary mucinous neoplasm
LIF	Leukemia inhibitory factor
LIFR	Leukemia inhibitory factor receptor
LPC	Lysophosphatidylcholine
LRRC15	Leucine-rich repeat containing 15
MAPK	Mitogen-activated protein kinase
MCP-1	Monocyte chemoattractant protein-1
M-CSF	Macrophage-colony stimulating factor
MCT1	Monocarboxylate transporter 1
MCT4	Monocarboxylate transporter 4
MDSCs	Myeloid-derived suppressor cells
MHC	Major histocompatibility complex
miRNA	microRNA
MMP9	Matrix metallopeptidase 9
MSCs	Mesenchymal stem cells
myCAFs	myofibroblastic CAFs
NF-κB	Nuclear factor kappa-light-chain-enhancer of activated B cells
NGF	Nerve growth factor
NI	Neural invasion
NK cells	Natural killer cells
OTI	MHC class I-restricted ovalbumin-specific T cell receptor
OTII	MHC class II-restricted ovalbumin-specific T cell receptor
OVA	Ovalbumin
PanIN	Pancreatic intraepithelial neoplasia
PBMCs	Peripheral blood mononuclear cells
PD-1	Programmed death-1
PD-L1	Programmed death-ligand 1
PDAC	Pancreatic ductal adenocarcinoma
PDGF	Platelet-derived growth factor
PDGFRα	Platelet-derived growth factor receptor-alpha
PDGFRβ	Platelet-derived growth factor receptor-beta
PDPN	Podoplanin
Pdx1	Pancreatic and duodenal homeobox 1
PKT	Ptf1a-Cre;LSL-Kras^G12D/+^;Tgfbr2^flox/flox^
PODXL	Podocalyxin
PSCs	Pancreatic stellate cells
PTCH	Patched
PTF1α	Pancreas associated transcription factor 1-alpha
RCP	Rab-coupling protein
ROS	Reactive oxygen species
SAA3	Serum amyloid A3
scRNA-seq	Single cell RNA-sequencing
SHH	Sonic hedgehog
SMAD4	Mothers against decapentaplegic homolog 4
SMO	Smoothened
SOCS1	Suppressor of cytokine signaling 1
ST2	Suppression of tumorigenicity 2
STAT3	Signal transducer and activator of transcription 3
TAMs	Tumor-associated macrophages
TCA	Tricarboxylic acid
TCR	T-cell receptor
TGF-β	Transforming growth factor-beta
TGFBI	Transforming growth factor-beta-induced
TGFBR2	Transforming growth factor-beta receptor 2
TME	Tumor microenvironment
TNF-α	Tumor necrosis factor-alpha
Tregs	Regulatory T cells
VEGF	Vascular endothelial growth factor
YAP1	Yes-associated protein 1
